# A practical algorithmic approach to mature aggressive B cell lymphoma diagnosis in the double/triple hit era: selecting cases, matching clinical benefit: A position paper from the Italian Group of Haematopathology (G.I.E.)

**DOI:** 10.1007/s00428-019-02637-2

**Published:** 2019-08-06

**Authors:** Arianna Di Napoli, D. Remotti, C. Agostinelli, M. R. Ambrosio, S. Ascani, A. Carbone, F. Facchetti, S. Lazzi, L. Leoncini, M. Lucioni, D. Novero, S. Pileri, M. Ponzoni, E. Sabattini, C. Tripodo, A. Zamò, M. Paulli, L. Ruco

**Affiliations:** 1 Pathology Unit, Department of Clinical and Molecular Medicine, Sant’Andrea Hospital, Sapienza University, Via di Grottarossa 1035, 00189 Rome, Italy; 2 Pathology Unit, San Camillo-Forlanini Hospital, Rome, Italy; 3 Hematopathology Unit, S. Orsola University Hospital, Bologna, Italy; 4 Pathology Unit, Department of Medical Biotechnology, University of Siena, Siena, Italy; 5 Pathology Unit, Ospedale di Terni, University of Perugia, Terni, Italy; 6 Department of Pathology, Centro di Riferimento Oncologico di Aviano, Istituto di Ricovero e Cura a Carattere Scientifico, Aviano, Italy; 7 Pathology Section, Department of Molecular and Translational Medicine, University of Brescia, Brescia, Italy; 8 Pathology Unit, University of Pavia and Fondazione IRCCS San Matteo Policlinico, Pavia, Italy; 9 Department of Oncology, University of Turin and Pathology Unit, AOU Città della Salute e della Scienza, Turin, Italy; 10 Division of Haematopathology, European Institute of Oncology, Milan, Italy; 11 Ateneo Vita-Salute, Pathology Unit, IRCCS San Raffaele Scientific Institute, Milan, Italy; 12 Tumor Immunology Unit, Department of Health Sciences, University of Palermo, Palermo, Italy; 13 Tumor and Microenvironment Histopathology Unit, the FIRC Institute of Molecular Oncology (IFOM), Milan, Italy; 14 Italian Group of Haematopathology (GIE), Rome, Italy; 15 Pathology Board of the Italian Lymphoma Foundation (FIL), Rome, Italy

**Keywords:** HGBL, Double hit, DLBCL, Diagnosis, FISH, MYC

## Abstract

An accurate diagnosis of clinically distinct subgroups of aggressive mature B cell lymphomas is crucial for the choice of proper treatment. Presently, precise recognition of these disorders relies on the combination of morphological, immunophenotypical, and cytogenetic/molecular features. The diagnostic workup in such situations implies the application of costly and time-consuming analyses, which are not always required, since an intensified treatment option is reasonably reserved to fit patients. The Italian Group of Haematopathology proposes herein a practical algorithm for the diagnosis of aggressive mature B cell lymphomas based on a stepwise approach, aimed to select cases deserving molecular analysis, in order to optimize time and resources still assuring the optimal management for any patient.

## Introduction

Diffuse large B cell lymphomas not otherwise specified (DLBCL NOS) represent a spectrum of malignancies associated with diversified clinical outcomes. Characterization of molecular features of clinical importance, such as the cell of origin (COO) and the rearrangements of *MYC*, *BCL2*, and *BCL6* genes, has been incorporated as a new requirement in the revised World Health Organization (WHO) classification of tumors of hematopoietic and lymphoid tissues [1].

**Fig. 1:**
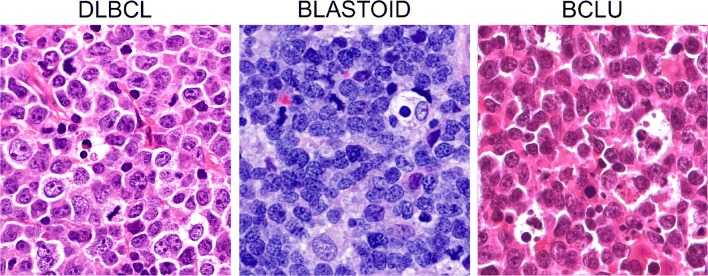
Morphological features of aggressive mature B cell lymphomas. In DLBCL, the cells are pleomorphic with centroblastic and/or immunoblastic features. Blastoid cells are medium-sized cells with a fine chromatin pattern and inconspicuous nucleoli. Cases with features overlapping between BL and DLBCL (BCLU) show medium-sized cells, less monomorphic than in classical BL, with multiple paracentrally located nucleoli and frequent starry sky pattern

**Fig. 2:**
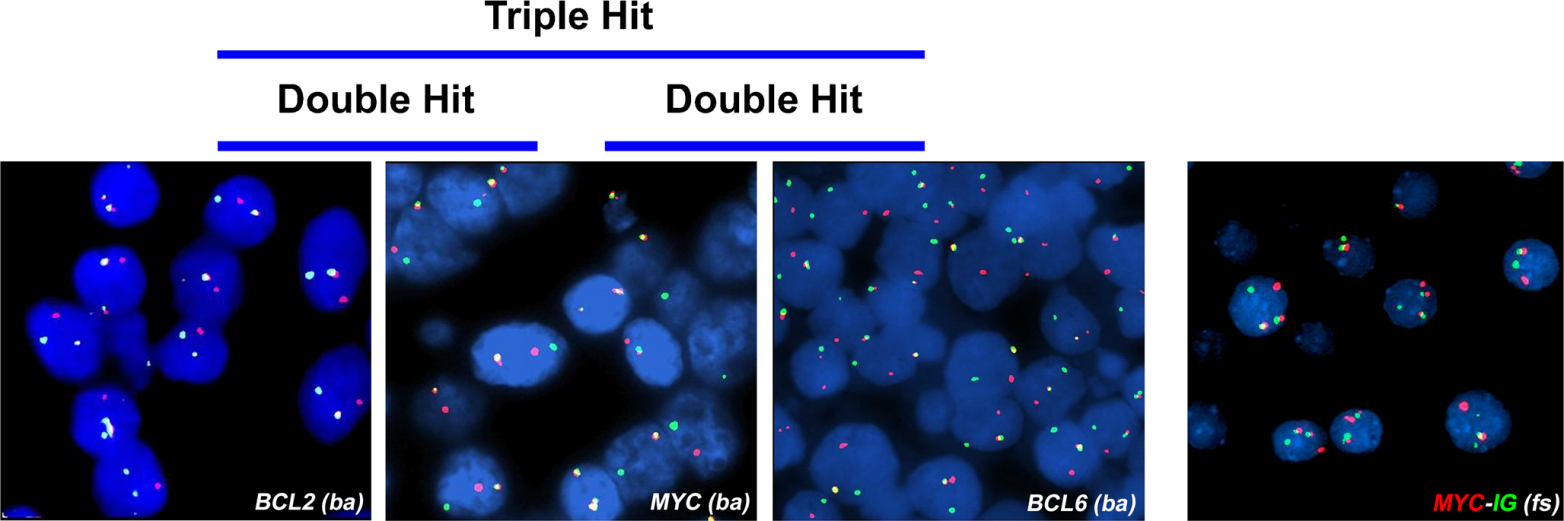
Interphase FISH showing rearrangements of *MYC*, *BCL2*, and *BCL6* genes using dual color break-apart (BA) probes. *MYC/IGH* translocation is detected using a dual color dual fusion FISH probe

Gene expression profiling (GEP) or surrogated immunohistochemical algorithms allow subclassification of DLBCL NOS mainly into the germinal center (GCB) and the activated (ABC) or non-GCB types based on the cell of origin, with ABC lymphomas displaying poorer prognosis than GCB ones [2]. pt?>Fluorescence in situ hybridization (FISH) is required to distinguish among high-grade B cell lymphomas with double or triple hit rearrangement (HGBL DH/TH), high-grade B cell lymphomas not otherwise specified (HGBL NOS), and DLBCL NOS. HGBL DH/TH are aggressive mature B cell lymphomas with variable morphology, ranging from pleomorphic large cells to medium-sized cells with features intermediate between DLBCL and Burkitt lymphoma (BCLU), to blastoid cells (Fig. 1), where FISH analyses identify *MYC* gene rearrangement in association with *BCL2* and/or *BCL6* gene rearrangements (Fig. 2). Notably, HGBL DH/TH account for approximately 5% of all cases with DLBCL morphology and generally have a low complete response rate with R-CHOP that advises for more intensive chemotherapy regimens [1, 3]. HGBL NOS includes cases with neoplastic B cells having either blastoid morphology or histopathological features intermediate between DLBCL and Burkitt Lymphoma (BCLU) that do not carry a double or a triple rearrangement. Recently, gene expression signatures and mutational profiles identified high-risk patients with DLBCL comprising double hit lymphomas [4].

GEP and FISH studies, which are the elective technologies for the definition of the COO, of gene rearrangements and of high-risk lymphomas are expensive, time consuming, and not available in all laboratories. To date, no guidelines are available driving both pathologists and clinicians in the selection of aggressive mature B cell lymphomas deserving molecular analyses in a cost-effective management of the patients. The use of immunohistochemistry, cheaper and widely applicable, as surrogate tool for the assessment of the COO and the presence of *MYC* gene rearrangements in DLBCLs have been proposed [5–8]. However, this approach bears intrinsic limitations. Although HGBL-DH overexpress MYC and BCL2 proteins in most instances, they only account for a small proportion of the so-called double expressors DLBCL (DE DLBCL). In addition, HGBL carrying *MYC* and *BCL2* gene rearrangements almost exclusively belong to the GCB category, while the majority of DE DLBCL fall into the non-GCB group [3, 8]. Of note, nearly 20% of GCB DLBCL carrying *MYC* rearrangement do not express MYC protein [8].

On the other hand, molecular subtyping all DLBCLs to identify all HGBL DH/TH may be unnecessary, since treatment choice is also driven by patient’s age, comorbidities, and performance status, with dose-intense treatment options being usually reserved to fit and young (age < 60 years) patients.

The purpose of this article is to propose a stepwise, working algorithm aimed at the rationalization of the diagnostic efforts in aggressive mature B cell lymphomas. The attempt is to provide minimal required criteria to select cases deserving FISH analysis, in order to save time and resources still assuring the optimal management for any patient.

## Sample requirements

This diagnostic workflow applies to any nodal/extranodal, aggressive mature B cell lymphoma that does not fulfill the diagnostic criteria of any specific DLBCL entity (e.g., EBV+ DLBCL, primary mediastinal B cell lymphoma, primary central nervous system DLBCL, T cell histiocyte-rich DLBCL) as recommended by the revised version of the World Health Organization classification of lymphoid malignancies.

In order to enable all the required immunohistochemical and molecular analyses and avoid pre-analytical biases, samples should contain an adequate amount of tissue embedded in paraffin within 24 h of formalin fixation. Since core needle biopsies might not be fully informative to render an accurate diagnosis of lymphoma, excisional lymph node biopsies should be favored whenever possible.

## Diagnostic workflow

An initial diagnosis of an aggressive mature B cell lymphoma should incorporate the assessment of cytological and immunohistochemical features, including the COO, and the percentage of MYC- and BCL2-expressing cells. Whenever a B cell lymphoma with a DLBCL morphology displays either a GCB COO and/or a double expression of MYC and BCL2 proteins (in more than 40% and 50% of neoplastic cells, respectively) (DE DLBCL), analysis of *MYC*, *BCL2*, and *BCL6* gene rearrangements by FISH is indicated in order to rule out the possibility of a HGBL DH/TH. Although there is no complete agreement about the percentage of MYC protein-expressing cells that accurately predicts the presence of *MYC* gene rearrangement [6, 9, 10], the cutoff value of 70% has been recently reported to be reproducible among different centers and of clinical value in identifying patients with a worse prognosis [7].

Before proceeding with FISH analysis, in DLBCL cases, it is highly recommended to discuss upfront with the referring hematologist the results of immunohistochemical screening in order to verify patient’s fitness and potential eligibility to undergo intensified therapy for HGBL DH/TH.

**Fig. 3:**
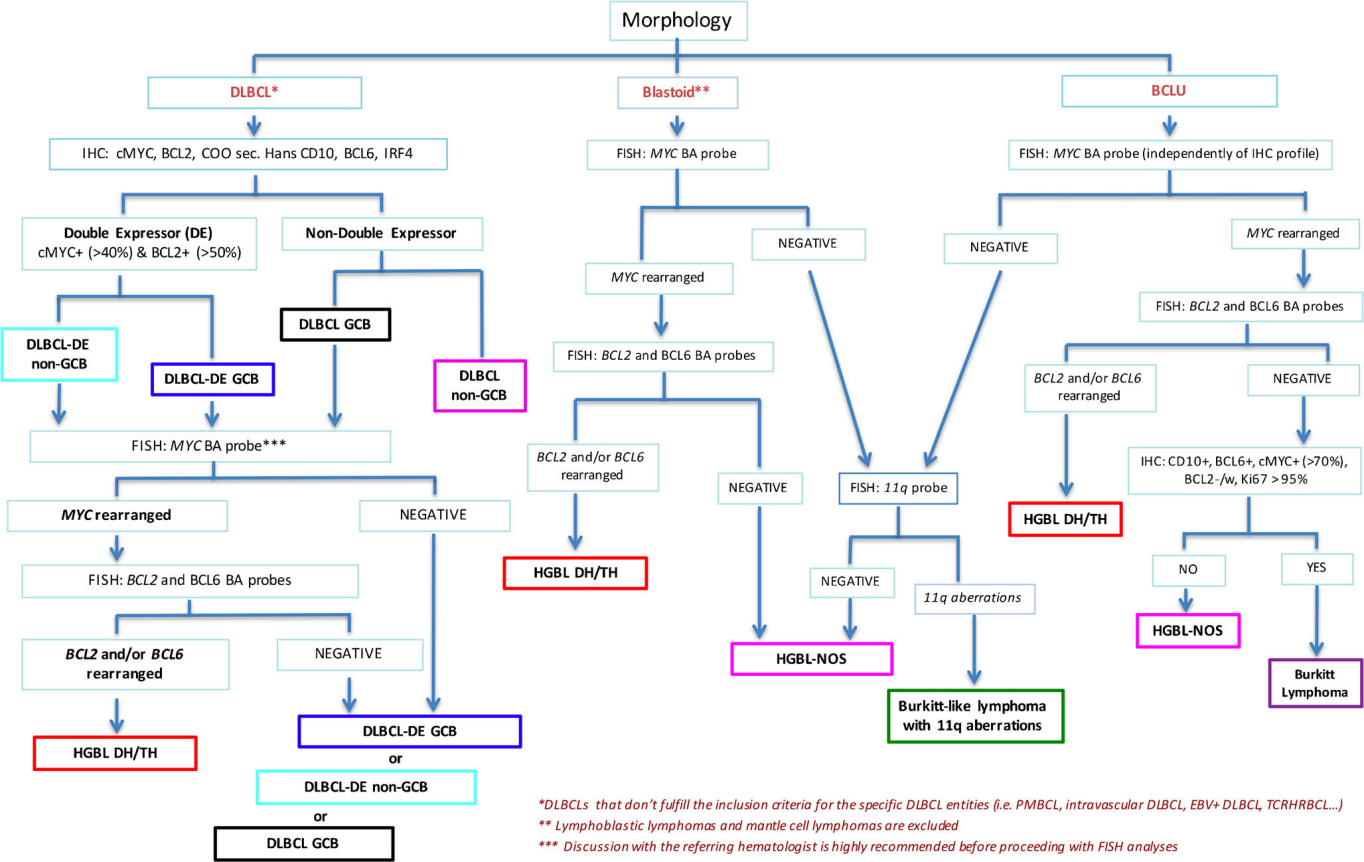
Diagnostic workflow for the diagnosis of aggressive mature B cell lymphomas . The workflow applies to DLBCLs that don’t fulfill the inclusion criteria for the specific DLBCL entities (i.e. primary mediastinal B cell lymphoma (PMBCL), intravascular DLBCL, EBV+ DLBCL, T cell rich histiocyte rich B cell lymphoma (TCRHRBCL), etc (*) and to blastoid lymphomas excluding lymphoblastic lymphomas and mantle cell lymphomas (**). In DLBCL discussion with the referring hematologist is highly recommended before proceeding with FISH analyses (***).

On the contrary, FISH analyses should be performed in any case of:

- B cell lymphomas with blastoid morphology, with the exclusion of TdT+ lymphoblastic lymphoma or cyclin D1+ pleomorphic/blastoid mantle cell lymphoma (Fig. 3). In these cases, FISH analysis for *MYC*, *BCL2*, and *BCL6* genes allows classification of the malignancy as HGBL DH/TH or HGBL NOS. *CCDN1* translocations should also be investigated to rule out cyclin D1-expressing DLBCL.
- B cell lymphomas with morphological features intermediate between DLBCL and Burkitt lymphoma (BCLU). In these cases, independently of the immunophenotype exhibited by tumor cells, *MYC*, *BCL2*, and *BCL6* rearrangements should be investigated for the differential diagnosis among HGBL DH/TH, HGBL NOS, and Burkitt lymphoma (Fig. 3). In cases without *MYC* translocation, FISH analysis for chromosome 11q is required to identify Burkitt-like lymphomas with 11q aberrations.

## Immunohistochemistry

Aggressive mature B cell lymphomas should express B cell–associated antigens (e.g., CD20, CD19, CD79a) and lack cyclin D1. In the case of a cyclin D1+ large B cell lymphoma, immunohistochemistry for CD5, SOX11, and FISH analysis with a *CCND1* break-apart probe must be performed in order to rule out a pleomorphic/blastoid mantle cell lymphoma [1]. The use of CD5 is also encouraged to identify de novo CD5+ DLBCLs, which might display an unfavorable outcome [11].

The COO of DLBCL can be investigated by gene expression profiling (GEP) or, alternatively, by immunohistochemistry (IHC) following algorithms suggested by the 2017 WHO Classification [1]. Among these, the most popular is Hans algorithm, which splits DLBCLs in germinal center (GCB) and non-germinal center (non-GCB) type based on the expression of CD10, BCL6, and IRF4/MUM1 proteins [12]. Its output shows reasonable correlation with the GEP, although some cases of DLBCL GCB type are misclassified as non-GCB type by IHC [5]. In addition to its role in discriminating different DLBCL prognostic subgroups (non-GCB carrying worse prognosis in comparison with GCB type), determination of COO might help in identifying those cases potentially harboring rearrangements of *MYC*, *BCL2*, and *BCL6.* Indeed, almost all the HGBL DH/TH fall within the GCB subtype with less than 1% of ABC harboring *MYC* and *BCL2* and 2% *MYC* and *BCL6* rearrangements [3].

Immunohistochemical investigation of MYC and BCL2 protein expression in DLBCL is highly recommended since overexpression of these proteins is associated with shorter survival [10, 13, 14]. Moreover, HGBL DH without MYC or BCL2 overexpression display a more favorable outcome than double expressor HGBL DH [7, 10, 13, 14]. Cutoff values for MYC and BCL2 that have been significantly associated with survival are 40% and 50%, respectively (independently of the intensity of the staining) [9]. Whenever the IHC staining is not homogeneously distributed across the section, the percentage of positive cells should be calculated as the average, and the occurrence of hot spots with MYC > 70% should be reported. A high percentage of MYC+ cells is more likely to be associated with MYC translocation [6, 7, 9]. Some pathologists have advocated the use of Ki67 staining, although the proliferative fraction is variable in HGBL DH/TH and it cannot be considered a reliable marker for screening patients that require FISH [1, 10].

## FISH analysis

Rearrangements of *MYC*, *BCL2*, and *BCL6* genes are generally assessed using break-apart probes. Since the definition of HGBL DH/TH requires the presence of *MYC* rearrangement, this could be investigated first, followed by *BCL2* and *BCL6* gene analyses in *MYC* rearranged cases. The use of dual color dual fusion *IGH-MYC* probes in addition to *MYC* break-apart probes (Fig. 2) increases the sensitivity of detection [14]. Furthermore, the definition of the partner gene (*IGH* or non-*IGH*) of *MYC* translocation could be clinically relevant, although this issue is still debated [9, 14, 15]. By an administrative standpoint, FISH analysis could be requested as an additional investigation either by the clinician or by the pathologist according to local rules.

## Concluding remarks

In conclusion, we believe that the application of the proposed workflow could represent a useful strategy to rationalize the procedures and optimize the resources, speeding up the diagnosis of aggressive mature B cell lymphomas and allowing the more appropriate treatment option for each patient.
